# Anti‐angiogenic effect of quercetin and its 8‐methyl pentamethyl ether derivative in human microvascular endothelial cells

**DOI:** 10.1111/jcmm.14455

**Published:** 2019-08-01

**Authors:** Gabriella Lupo, Maria Teresa Cambria, Melania Olivieri, Concetta Rocco, Nunzia Caporarello, Anna Longo, Guido Zanghì, Mario Salmeri, Mario C. Foti, Carmelina Daniela Anfuso

**Affiliations:** ^1^ Section of Medical Biochemistry, Department of Biomedical and Biotechnological Sciences (Biometec), School of Medicine University of Catania Catania Italy; ^2^ Institute of Biomolecular Chemistry of CNR Catania Italy; ^3^ Department of Surgery (CHIR), School of Medicine University of Catania Catania Italy

**Keywords:** 6,8‐dibromoquercetin, 8‐methylquercetin pentamethyl ether, human retinal endothelial cells, quercetin, quercetin dimer, tumour angiogenesis, VEGFR‐2

## Abstract

Angiogenesis is involved in many pathological states such as progression of tumours, retinopathy of prematurity and diabetic retinopathy. The latter is a more complex diabetic complication in which neurodegeneration plays a significant role and a leading cause of blindness. The vascular endothelial growth factor (VEGF) is a powerful pro‐angiogenic factor that acts through three tyrosine kinase receptors (VEGFR‐1, VEGFR‐2 and VEGFR‐3). In this work we studied the anti‐angiogenic effect of quercetin (Q) and some of its derivates in human microvascular endothelial cells, as a blood retinal barrier model, after stimulation with VEGF‐A. We found that a permethylated form of Q, namely 8MQPM, more than the simple Q, is a potent inhibitor of angiogenesis both in vitro and ex vivo. Our results showed that these compounds inhibited cell viability and migration and disrupted the formation of microvessels in rabbit aortic ring. The addition of Q and more significantly 8MQPM caused recoveries or completely re‐establish the transendothelial electrical resistance (TEER) to the control values and suppressed the activation of VEGFR2 downstream signalling molecules such as AKT, extracellular signal‐regulated kinase, and c‐Jun N‐terminal kinase. Taken together, these data suggest that 8MQPM might have an important role in the contrast of angiogenesis‐related diseases.

## 1 INTRODUCTION

In the human retina, homeostasis is maintained by the blood retinal barrier (BRB). The retinal continuous endothelium forms the main structure of the BRB and leans on a basal lamina which is covered by the processes of astrocytes, Müller cells and pericytes. These cellular types all contribute to the maintenance of the BRB,^1^ whose integrity is essential for proper vision. Physiologically, the intercellular spaces among the BRB endothelial cells are sealed by complex tight junctions and the cells themselves lack fenestrations and have few pinocytotic vesicles. These features result in the typical restricted paracellular permeability and high transendothelial electrical resistance (TEER). The BRB breakdown greatly contributes to the pathology and vision loss in retinal disorders such as uveitis, diabetic retinopathy, age‐related macular degeneration and tumour.^2^

Growth factors, such as vascular endothelial growth factor (VEGF) and pro‐inflammatory cytokines have been implicated in the pathophysiology of these diseases and contribute to the clinically observed retinal inflammation, angiogenesis and vascular hyper‐permeability.^3^, ^4^ The treatment with anti‐VEGF agents significantly reduces the abnormal growth of microvessels.^5^ Clinical trials have demonstrated the efficacy of these treatments in the advanced stages of neovascular eye diseases hence confirming the modulatory role of VEGF in the pathogenesis and progression of retinal aberrant neovascularization, which occurs in pathological conditions,^6^ diabetic retinopathy,^7^ and tumour.^8^ The permeability‐ and proangiogenic‐inducing effects of VEGF on endothelial cells are mainly mediated by VEGF receptor‐2 (VEGFR‐2), whereas VEGFR‐1, in dependence of the tissue context, is both a negative and positive regulator of VEGFR‐2 signalling. Activation of VEGFR‐2 contributes to phosphorylation of multiple downstream signals such as ERK (extracellular signal‐regulated), AKT (also termed protein kinase B), JNK (c‐Jun N‐terminal kinase), which cause proliferation, migration and tube formation of endothelial cells.^9^, ^10^

**Scheme 1:**
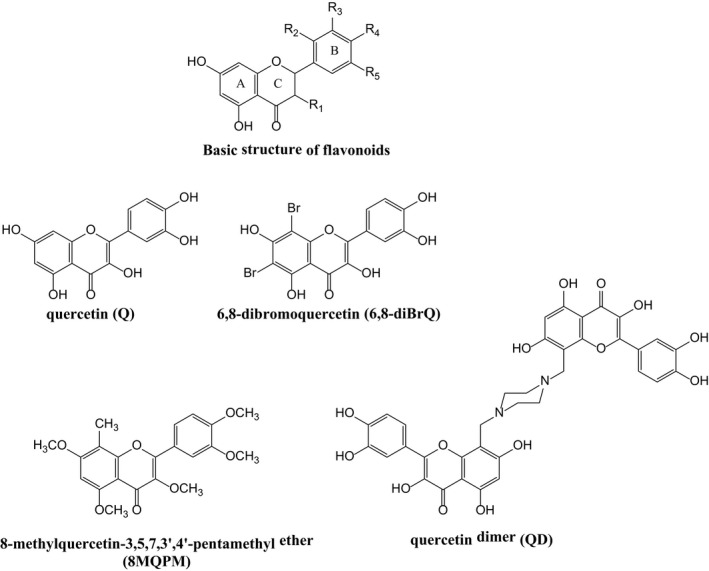
Compounds tested in this study. The acronyms of their chemical names are indicated in parentheses

Quercetin (Q) is one of the most widely diffused flavonoids in fruits and vegetables,^11^ usually present in the form of 3‐*O*‐glycoside with the d‐glucose, galactose or rhamnose. It is an antioxidant and free radical scavenger,^12^ and it has been shown to have anti‐inflammatory 13 and neuroprotective^14^ effects. The inhibition of certain enzymes involved in proliferation and signal transduction pathway including tyrosine kinase, protein kinase C,^15^ PI‐3 kinase,^16^ proline‐directed protein kinase fatty acid in human prostate carcinoma cells^17^ and JNK^18^ and the induction of apoptosis and growth inhibition in lung cancer cells^19^ by Q have already been highlighted. Moreover, there exists a considerable number of data reporting on the capability of Q to induce cell cycle arrest and apoptosis and to being an inhibitor of carcinogenesis and angiogenesis.^20^, ^21^ Several studies have highlighted that the anti‐cancerous property of Q is due to the down‐regulation of the VEGF and Hypoxia‐inducible factor‐1 (HIF‐1) expression.^22^ It has been shown that Q has negative^23^ as well as positive effects^24^ on HIF‐1 and VEGF expressions in different types of cell and, to date, several uncertainties have been raised regarding its absorption and availability in tumour. It has been demonstrated that Q inhibits angiogenesis by targeting VEGFR‐2 regulated AKT/mTOR/P70S6K signalling pathway.^25^ In previous experiments, we have shown that the tumour angiogenic stimulus is driven through the phosphorylation/activation of Protein kinase C α, ERK1/2 and cytosolic calcium‐dependent and ‐independent phospholipases A_2_, all events required for cell proliferation and motility.^26^, ^27^, ^28^ As mentioned above, several studies have confirmed that Q, likewise other common dietary flavonoids, inhibits the angiogenic process both in vitro and in vivo.^25^ On the other hand, the derivatives of Q often manifest different effects on the activation of signalling pathways and in their consequent biological effects, for example angiogenesis. Unlike quercetin‐3′‐sulphate, for instance, quercetin‐3‐*O*‐glucuronide (Q3GA), suppresses the in vivo VEGF‐induced angiogenesis through ERK inhibition.^29^ Quercetin has long received great interest in these studies but its poor bioavailability and low stability in aqueous media definitely limit the use of this flavonoid in clinical applications. For these reasons, in this study three derivatives of Q, namely, 6,8‐dibromoquercetin (6,8‐diBrQ), 8‐methylquercetin pentamethyl ether (8MQPM) and quercetin dimer (QD; see Scheme 1) were prepared and tested along with Q as therapeutic agents. Quercetin was permethylated in order to improve its ability to cross the cell membranes. On the other hand, the bromo derivative (6,8‐diBrQ) is able to release small quantities of bromine in solution^30^ which may produce interesting and useful effects on cells. In this work, human primary endothelial cells, isolated from retinal microcapillaries (HREC) as a BRB model‐system, were used for the experiments. Human retinal endothelial cells were treated with conditioned medium (CM) from Y‐79 human retinoblastoma cell line or were VEGFA‐stimulated in order to reproduce the angiogenic environment of the above‐mentioned retinal diseases and cancer. Our findings reveal that Q and even more so 8MQPM offer protection against the powerful pro‐angiogenic stimulus present in our in vitro human BRB model.

## 2 MATERIALS AND METHODS

### 2.1 Chemicals

All reagents and solvents for the synthesis of 8MQPM were purchased from Sigma‐Aldrich and were used without further purification. The ^13^C and ^1^H Nuclear magnetic resonance spectroscopy (NMR) spectra were recorded at 400.13 MHz (^1^H) and 100.62 MHz (^13^C) in CDCl3 solutions at 298 K on a Bruker AvanceTM 400 spectrometer. Electrospray ionization mass spectrometers (ESI‐MS) spectra were recorded with a GC‐MS QP5050A Shimadzu spectrometer.

### 2.2 Synthesis of quercetin derivatives

Quercetin dihydrate (HPLC) ≥98%, 6, 8‐diBrQ and QD were available from previous studies. The syntheses of 6, 8‐diBrQ and QD along with the NMR spectra are reported in references^31^ and 32, 33 respectively. The synthesis of 8MQPM and its carbon and proton NMR spectra are instead reported below.

One gram of quercetin dihydrate (3.0 mmol) was solubilized in a mixture of 60 mL of Tetrahydrofuran and 5 mL of Dimethylformamide. Then, 6.5 g (20 mmol) of Cs_2_CO_3_ and 3 mL (48 mmol) of CH_3_I were added and the mixture was heated at reflux under stirring for 5 hours. After cooling, the mixture was filtered and then was treated with a solution of 0.5 N HCl. The aqueous mixture was extracted three times with 100 mL portions of CH_2_Cl_2_. The organic phase was washed with a NaHCO_3_ solution and then dried over MgSO_4_. The solvent was removed and the crude residue was purified over silica gel (40‐63 μm) using a gradient (40%–100%) of ethyl acetate in *n*‐hexane to yield 153 mg of 8MQPM corresponding to a final yield of ca. 13%. The compound was identified through its ^1^H and ^13^C NMR spectra in CDCl_3_ at 298 K. ^1^H NMR (CDCl_3_): *δ* = 2.20 (s, 3H, CH_3_), 3.87 (s, 3H, OCH_3_), 3.91 (s, 3H, OCH_3_), 3.93 (s, 3H, OCH_3_), 3.98 (s, 6H, two OCH_3_), 6.70 (s, 1H, H‐6), 6.99 (d, J = 8 Hz, 1H, H‐5′), 7.72 (m, 2H, H‐6′and H‐2′). ^13^C NMR (CDCl_3_): *δ* = 8.03 (CH_3_), 55.82, 55.94, 59.77 and 61.43 (five OCH_3_), 94.57 (C‐6), 110.72 (C‐5′), 111.06 (C‐2′), 112.33 (C‐10), 118.00 (C‐8), 121.57 (C‐6′), 123.33 (C‐1′), 140.97(C‐3), 148.59 (C‐3′), 150.75 (C‐4′), 152.91 (C‐2), 156.30 (C‐9), 157.46 (C‐5), 162.19(C‐7), 173.34 (C‐4). The ESI‐MS of 8MQPM (MeOH) in the positive ion‐mode showed a peak at m/z 387.1 [M + H]^+ ^confirming the structure of the derivative.

### 2.3 Reagents and antibodies

Recombinant human VEGF‐A (VEGF‐A165 isoform) was purchased from Peprotech (Rocky Hill, NJ, USA). Serum‐free endothelial cell basal medium (EBM) was from ScienceCell Research Laboratories (San Diego, CA, USA). Antibiotics and other reagents for cell culture were from Invitrogen Life Technologies. Rabbit polyclonal anti‐phospho‐VEGF Receptor 2 (phospho Y1054; ab5472) and mouse monoclonal anti‐VEGF Receptor 2 (ab9530) were purchased from Abcam (1:1000); rabbit polyclonal anti‐phospho‐Akt (Ser473) (#9271), rabbit polyclonal anti‐Akt (#9272), rabbit polyclonal anti‐phospho‐p44/42 (#9101) and rabbit polyclonal anti‐p44/42 MAPK (Erk1/2) (#9102) were purchased from Cell Signaling technology (1:1000); rabbit polyclonal anti‐p‐JNK (Thr 183/Tyr 185) (sc‐12882) and mouse monoclonal anti‐JNK (sc‐1648) were purchased from Santa Cruz Biotechnology (1:500); mouse monoclonal anti‐ß‐actin antibody (AC‐15) was purchased from ThermoFisher Scientific (1:2000).

### 2.4 Cell cultures

Human retinal endothelial cells (Innoprot, Elexalde Derio, Spain) were grown in monolayer in EBM supplemented with 5% (v/v) foetal bovine serum (FBS), 1% endothelial cell growth supplement (ECGS), 100 mg/mL streptomycin and 100 U/mL penicillin. The serum was reduced to 0.25% v/v in experiments involving serum‐starvation.

Human Y‐79 retinoblastoma cells were purchased from the Cell Factory‐IST (Genova, Italy) and cultured in suspension in RPMI 1640 medium supplemented with 15% FBS, 1% glutamine and 1% sodium pyruvate and maintained at 37°C in humidified atmosphere containing 5% CO_2_.

### 2.5 CM collection

Human Y‐79 retinoblastoma cells (0.45‐5 × 10^6^ cells/mL) were cultured in serum free medium at 37°C for 24 hours to obtain CM.^34^ Conditioned medium was collected, centrifuged at 1000× g for 5 minutes, filtered with 0.2 μm filter and stored at −80°C until use. Conditioned medium was used without any dilution.^26^

### 2.6 Cell viability and number

To quantify cell viability in endothelial cells, the 3(4,5‐dimethylthiazol‐2‐yl)‐2,5‐diphenyl tetrasodium bromide (MTT) assay was used (Chemicon, Temecula, CA). Cells were plated in 96 well plates at a density of 1 × 10^4^ cells/well and were grown in complete medium in the absence or presence of increasing Q and Q derivatives concentrations (ie 25, 50 and 100 μM) for 24 and 48 hours. After the treatments, the cells were incubated with MTT for 4 hours and then 100 μL dimethyl sulfoxide was added and the absorbance was read at 590 nm, as previously described.^35^ To test cell proliferation, the cells were incubated with 25 ng/mL VEGF‐A or CM, as positive controls, and with CM containing 25 μmol/L Q or its derivatives. Bromodeoxyuridine (BrdU [Abcam, Cambridge, UK]) was added 4 hours before the end of the 24‐hour incubation period. Cells were fixed, DNA was denaturated and BrdU content was assessed by using anti‐BrdU monoclonal antibody, following the manufacturer's instructions.

### 2.7 Wound healing assay

HREC were seeded in 24 well tissue culture plates to a final density of 1.5 × 10^4^ cells/well. Monolayers of confluent cells were scratched with a p200 pipet tip. The wounds were photographed at 40× using a phase‐contrast microscope. Cells were stimulated for 48 hours in the presence of 25 ng/mL VEGF‐A or CM (as positive controls) or they were treated with CM containing 25 μmol/L Q or Q derivatives. After the incubations, endothelial cells invading the wound were quantified by computerized analysis of the photographs. The numbers of cells toward the wounds were counted using Imagej software (Imagej 1.50e, National Institutes of Health, NIH, Bethesda, MD) and were expressed as percentage of control cells, as previously described.^26^

### 2.8 TEER of cell layer

Transendothelial electrical resistance was measured with the Millicell‐ERS system (MERS 000 01; Millipore AG, Volketswil, Switzerland) as previously described.^27^ Values were expressed as *ω *× cm^2^ and calculated by the formula: (the average resistance of experimental wells − the average resistance of blank wells] × 0.33 (the area of the transwell membrane).

### 2.9 Tube formation assay

The ability of cells to migrate and organize into capillary‐like structures was evaluated by using the Matrigel assay (BD, Franklin Lakes, NJ). Briefly, 24 hours before the experiment, cells (1 × 10^4^) were shifted to a medium containing 0.25% serum and then were suspended in 200 µL of the same medium containing Q or its derivatives (25 μmol/L) or in 200 µL retinoblastoma CM containing Q or 8MQPM (25 μmol/L). The mixtures were seeded in 96 well plates covered with polymerized growth factor‐reduced Matrigel matrix, incubated for 4 hours (37°C, 5% CO_2_) and photographed at 100× magnification using an inverted Leica DM IRB microscope equipped with a charge‐coupled device (CCD) camera, as previously described.^36^, ^37^

### 2.10 Aortic ring assay

Rings from rabbit aorta were obtained by cross‐sectioning the thoracic aorta of New Zealand white male rabbits (1 mm intervals). The rings were placed on the bottom of 24‐well plates, pre‐coated with 150 μL of growth factor‐reduced Matrigel. After 10 minutes, the wells were rinsed with 150 μL of retinoblastoma CM in the absence or in the presence of the compounds under test. The medium was changed three times a week, starting from day 2. The aortic rings were observed daily for signs of angiogenic sprouting which was measured by counting the length of neovessels sprouting out of the rings after 14 days.^35^

### 2.11 Immunoblots

After treatments, the cells were detached by scraping, collected by centrifugation and lysed as previously described.^38^ The membranes were incubated with primary antibodies (4°C, o/n) and then with secondary antibodies (1 hour, room temperature). The membranes, after anti p‐VEGFR2, VEGFR2, p‐AKT, AKT, p‐ERK and ERK antibodies, were successfully stripped and re‐probed for β‐actin, to test the equal protein loading. The same procedure was followed for p‐JNK and JNK. The immunocomplexes were detected by enhanced chemiluminescence reagent (Amersham).

### 2.12 Statistical analysis

Statistical significance between two groups was analysed by Student's *t* test. One‐way and two‐way ANOVA, followed by Tukey's post hoc test, were used for multiple comparisons. *P* values <0.05 were considered statistically significant.

## 3 RESULTS

### 3.1 Quercetin and its derivatives affect HREC viability

**Figure 1:**
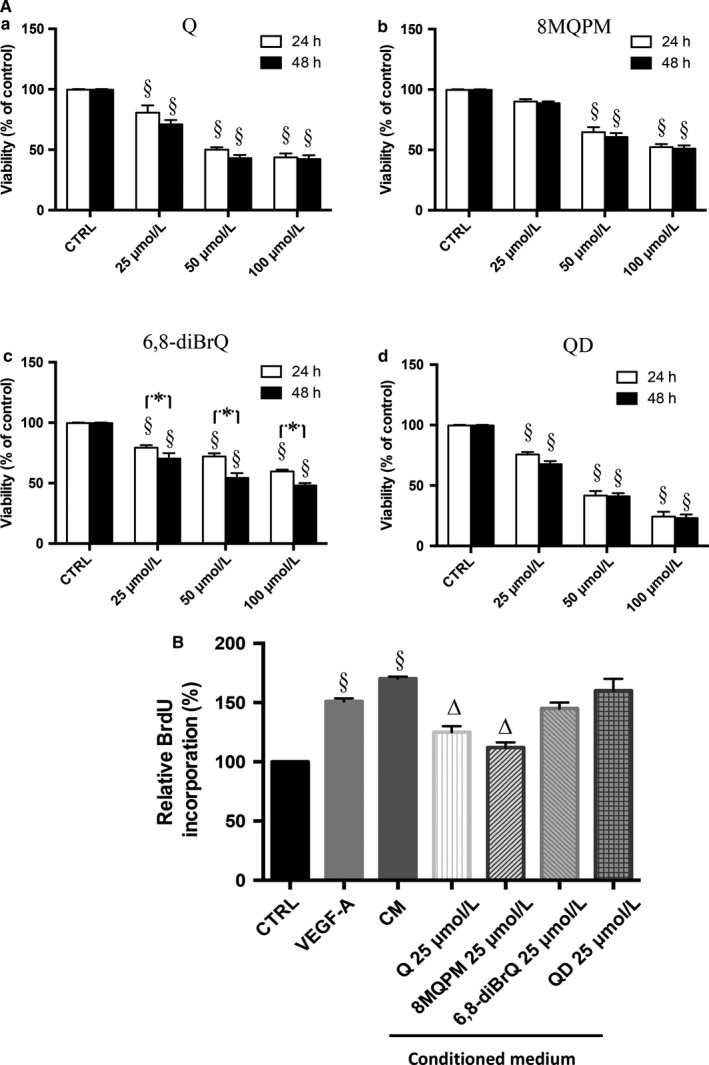
Effects of quercetin (Q) and its derivatives on human retinal endothelial cells (HREC) viability (A, 3(4,5‐dimethylthiazol‐2‐yl)‐2,5‐diphenyl tetrasodium bromide test) and proliferation (B, BrdU assay). A, a‐d, increasing doses (25‐50‐100 µmol/L) of quercetin and its derivatives were added to each well for 24 and 48 h. B, Effects of Q and its derivatives on retinoblastoma conditioned medium (CM)‐stimulated HREC proliferation. Values are expressed as the mean ± SD of three independent experiments (n = 3), ^§^ *P* < 0.01 versus control (CTRL); *P < 0.01, 24 h versus 48 h. B, effects of Q and its derivatives on retinoblastoma CM‐stimulated HREC proliferation. Values are expressed as the mean ± SD of three independent experiments (n = 3), ^§^ *P* < 0.01 versus CTRL; ^∆^ *P* < 0.01 versus CM‐stimulated HREC. One‐way ANOVA, followed by Tukey's test. Results are shown as per cent of control

In order to assess the toxicity of Q and of its derivatives dose‐ (25‐, 50‐ and 100 μmol/L concentrations in serum‐starved medium) and time‐dependent HREC viability were assayed by the MTT rapid colorimetric test, after 24 and 48 hours incubation (Figure 1). Quercetin and its derivatives caused a significant decrease in cell viability at the doses 50 and 100 μmol/L, both at 24 and 48 hours. At the concentration of 25 μmol/L, Q (panel A), 6,8‐diBrQ (panel C) and QD (panel D) caused a slight but significant decrease in the cell viability (Q, 20% and 29%, 6,8‐diBrQ, 21% and 30%, QD, 25% and 33% at 24 and 48 hours, respectively). On the contrary, no reduction in viability was observed with 8MQPM at 25 μmol/L after 48 hours incubation (panel B). This latter concentration was therefore used to perform the experiments here reported.

### 3.2 Quercetins and HREC proliferation

BrdU assay experiments were performed to investigate the cellular proliferative response after 24 hours incubation with Q and its derivatives (Figure 1 Panel E). Their effects on HREC proliferation in the presence of VEGF‐A or CM from retinoblastoma were examined in order to mimic an in vitro angiogenic stimulus.

The CM prompted a proliferative stimulus very similar to the incubation with VEGF‐A alone. The presence of both VEGF‐A at 25 ng/mL concentration and CM induced an expected increase in cell proliferation by 1.5‐ and 1.7‐fold, respectively. Interestingly, in the presence of CM, Q and 8MQPM caused a significant decrease in the HREC proliferation by 23% and 35% respectively, with respect to CM alone, bringing back the stimulated cells to almost the control proliferation levels. Unlike these ones, 6,8‐diBrQ and QD were not able to attenuate the induced angiogenic stimulus.

### 3.3 Quercetins and HREC migration

**Figure 2:**
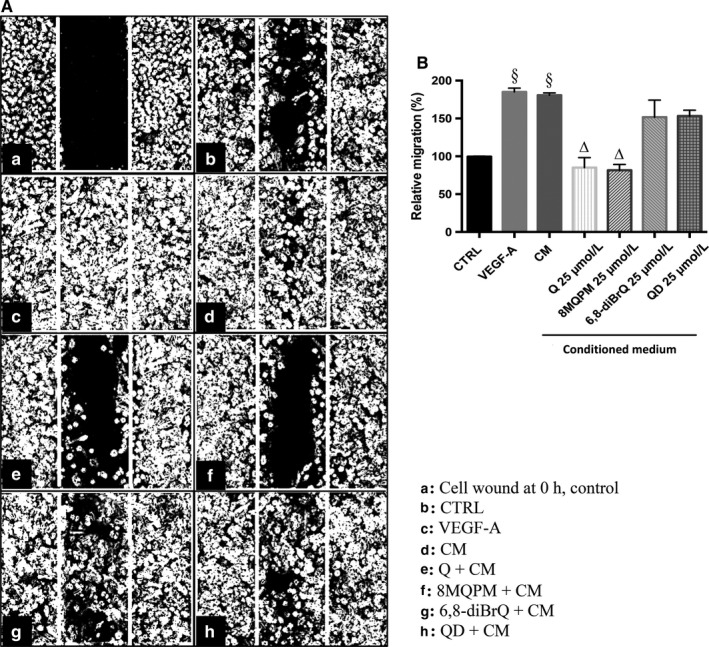
Effects of quercetin (Q) and derivatives on retinoblastoma conditioned medium (CM)‐stimulated on human retinal endothelial cells (HREC) migration. Contrast phase representative photographs show the scratch in the cell monolayer and the gradual wound closure after 48 h of incubation with vascular endothelial growth factor (VEGF)‐A (25 ng/mL) as positive control (c), CM (d) in the presence of Q, 8MQPM, 6,8‐diBrQ and QD (e, f, g and h, respectively) (panel A). The quantitative representation of the results as a percentage of wound closure by using Imagej software is shown (panel B). Values are expressed as the mean ± SD of three independent experiments (n = 3), ^§^ *P* < 0.01 versus control (CTRL); ^∆^ *P* < 0.01 versus CM‐stimulated HREC. One‐way ANOVA followed by Tukey's test. Results are shown as per cent of control

Endothelial cell migration is a critical process for wound healing and angiogenesis. The migration was evaluated by the wound‐healing assay (Figure 2). In panel A, contrast phase representative photographs of HREC cultures after wounding of the monolayers, and the wound edge advancement (from the white lines into the boxes) after 48 hours incubation, are reported. The box a shows the width of the scratch, immediately after being executed (time 0 hour, in the map legend), whereas the box b shows the HREC migration in starving serum‐medium (control, CTRL). The boxes c and d refer to cells incubated with VEGF‐A or CM, respectively; the effects of Q and of its derivatives on HREC are shown in boxes e, f, g, h. The quantitative representation of the results, expressing cell migration from the scratch border, is given as the ratio between the migration edge of treated samples versus the untreated control, arbitrarily posed at 100 (panel B). As expected from our previous data,^28^ both the growth factor and the retinoblastoma CM stimulated HREC motility so quantitatively similar by 1.8‐fold. Q significantly decreased such migration by 53% and even more so, 8MQPM by almost 55%, in the presence of CM. The 6,8‐diBrQ and QD reduced the migration by 17% and 15%, respectively, proving that their effect is not comparable to those of Q and 8MQPM. The individual compounds had no effect in the absence of VEGF‐A and CM (data not shown).

### 3.4 Effects of Q and its derivatives on blood‐retinal barrier: The TEER

**Figure 3:**
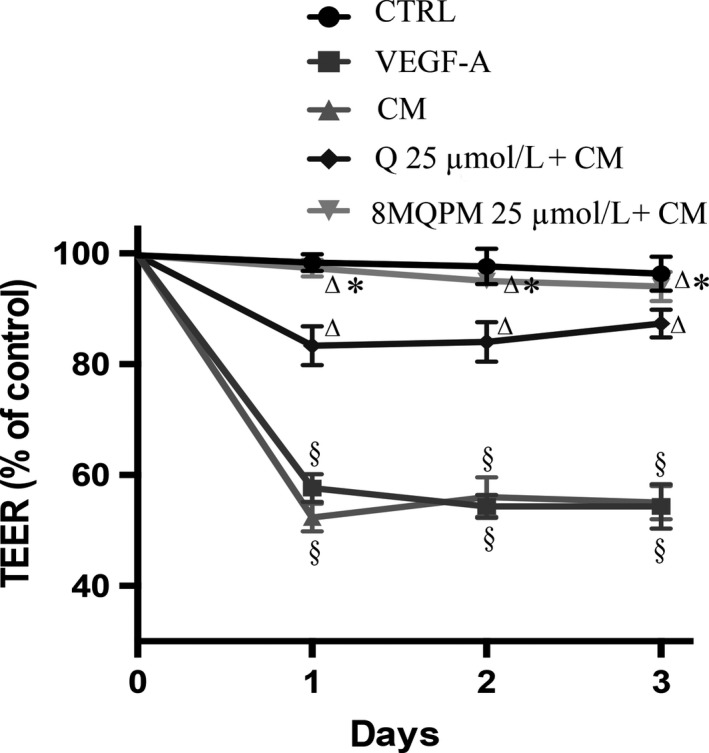
Quercetin (Q) and 8MQPM restore the TEER decrease in retinoblastoma conditioned medium (CM)‐stimulated human retinal endothelial cells (HREC). Values are expressed as the mean ± SD of three independent experiments (n = 3), ^§^ *P* < 0.01 versus control (CTRL); ^∆^ *P* < 0.01 versus CM‐stimulated HREC; ^*^ *P* < 0.01 versus Q. One‐way ANOVA followed by Tukey's test. Results are shown as per cent of control

The TEER represents the electrical resistance across the endothelial monolayer and is a sensitive and reliable method to confirm the barrier functional integrity. As well‐known, TEER is an important indicator of the molecular integrity of the tight junctions, which characterizes the strong lack of permeability through the BRB, so ensuring the physical structure required for the control of the incoming solutes towards the retinal neuropil. The effect of Q and 8MQPM on endothelial TEER is shown in Figure 3. As expected, VEGF‐A caused a significant decrease in TEER in a time‐dependent manner: 43% on the first day of incubation and 46% on the second and third day of incubation. Similar effects on the electrical resistance were also obtained by using CM from retinoblastoma. Q caused a significant recovery of TEER on the first, second and third day (approximately by 31%, compared to the treatment with CM). 8‐Methylquercetin pentamethyl ether almost completely re‐established the TEER values. These data show their strong protective effect against the collapse of the TEER determined by pro‐angiogenic growth factors on HREC. On the other hand, 6,8‐diBrQ and QD did not revert significantly the decrease in TEER induced by CM (data not shown).

### 3.5 Quercetin and 8MQPM inhibit angiogenesis by Matrigel assay

**Figure 4:**
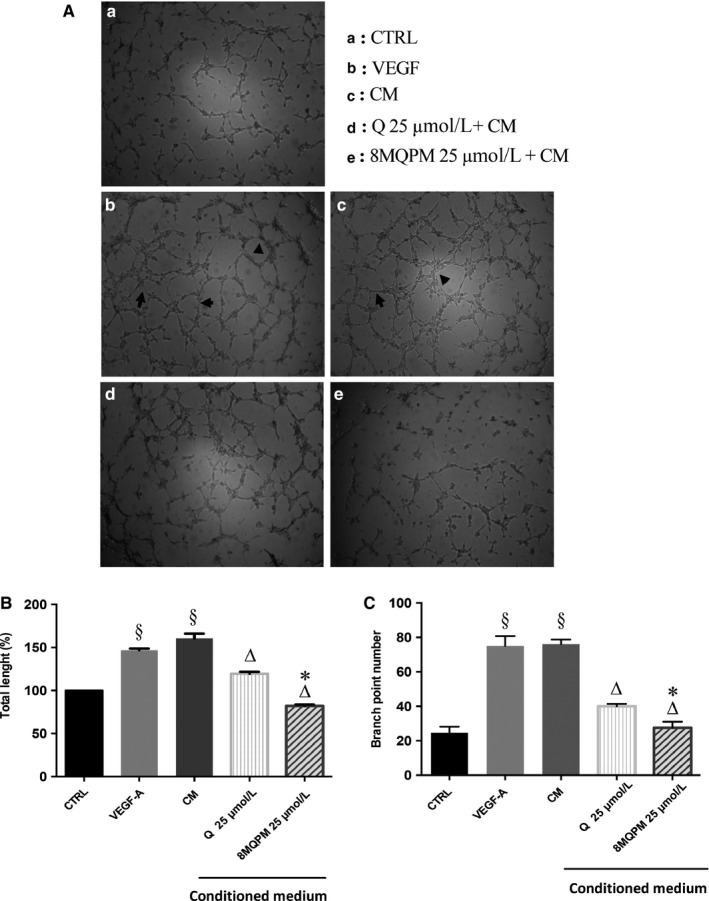
Quercetin (Q) and 8MQPM reduce the conditioned medium (CM)‐stimulated human retinal endothelial cells (HREC) tube formation on Matrigel. Representative optical phase‐contrast micrographs images are shown at 10X magnification (A). Tube elongation and branch points are showed by arrows and arrowheads, respectively. The quantification of tube length (B) and branch points (C) were calculated using Imagej software. Values are expressed as the mean ± SD of three independent experiments (n = 3), ^§^ *P* < 0.01 versus CTRL; ^∆^ *P* < 0.01 versus CM‐stimulated HREC; ^*^ *P* < 0.01 versus Q. One‐way ANOVA followed by Tukey's test. Results are shown as per cent of control for total length and branch point, respectively

Since an imbalance in the new blood vessel formation from those pre‐existing contributes to numerous infectious, inflammatory, immune, and, above all, malignant disorders,^39^ we examined the direct effect of Q and 8MQPM on angiogenesis by using the tube formation and the ex vivo rabbit aortic ring assays on Matrigel. The images in Figure 4, panel A, show representative optical contrast phase micrographs of proliferation and cell‐to‐cell organization at 24 hours incubation, highlighting that the tubular networks organized on Matrigel vary in the different environments. The number of interconnections among the tubes provides information on the way the HREC organize themselves and grow. Human retinal endothelial cells plated alone adhered on the matrix in a short time and expressed their typical elongated phenotype very quickly (Panel A, box a). Cell clusters emitted long offshoots from cell bodies. The emission of such processes (branch points) is essential for HREC in order to mutually come in physical contact, which is necessary for their recognition and spatial organization. In all culturing conditions, tube formation was measured by the tube length quantification and by counting the tubule branch points (arrows) defined as cell junctions containing at least three tubules (panels B and C). No significant differences were found, compared to control cells, after the incubation with Q and 8MQPM (data not shown). HREC adhesion and growth on Matrigel in VEGF‐A or CM conditioning environment were heavily magnified (panel A, boxes b and c). These angiogenic promoters induced the enhancements of both tube length (by 1.46‐ and 1.6‐fold, respectively) and branch points (by 3‐fold for both), in comparison to control unstimulated cells. The incubation of the cells with CM in the presence of Q significantly decreased the tube total length and the branch points number by 26% and 48%, respectively, compared to CM‐treatment (panel A, box d; panels B and C). The inhibition of the angiogenic parameters taken into account was strongly amplified by 8MQPM, which reduced the tube length by 49% and brought back the branch points number to control value (65% of reduction; panel A, box c; panels B and C).

### 3.6 Quercetin and 8MQPM inhibit angiogenesis in a rabbit aortic ring ex vivo model

**Figure 5:**
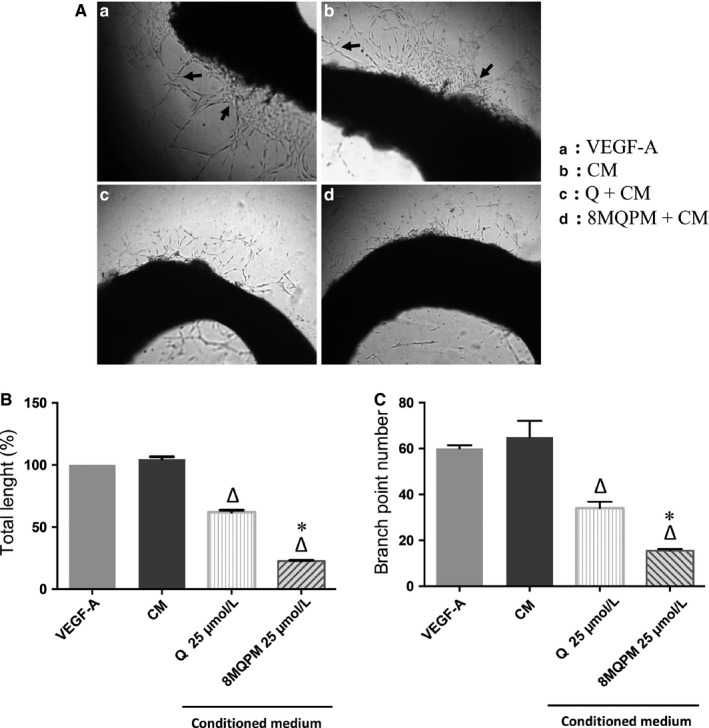
Effects quercetin (Q) and 8MQPM on retinoblastoma conditioned medium (CM)‐stimulated ex vivo rabbit aortic ring. Representative optical phase‐contrast micrographs images are shown at 10× magnification (panel A). Tube elongation (panel B) and branch points (panel C) were calculated using Imagej software. Values are expressed as the mean ± SD of three independent experiments (n = 3), ^∆^ *P* < 0.01 versus CM‐stimulated rings; ^*^ *P* < 0.01 versus Q. One‐way ANOVA followed by Tukey's test. Results are shown as per cent of control for total length

In the ex vivo rabbit aortic ring assay,^40^ the aortic extremely thin‐circular sections are embedded in biomatrix gels and cultured under chemically defined conditions. These sections generate arborizing vascular outgrowths which can be stimulated or inhibited by angiogenic regulators. Figure 5 (panel A) shows the production of endothelial sprouting in 10 days of incubation of the ring. In the absence of angiogenic stimulus or in the presence of Q or 8MQPM, a small production of endothelial sproutings was observed (data not shown). An evident expansion and organization of endothelial tubes from aorta rings was evident when the incubations were carried out in the presence of VEGF‐A or CM (panel A: arrows, boxes a and b, respectively). In these conditions the incubation of the cells with CM in the presence of Q (panel A, box c) decreased significantly the tube total length and the branch points number by 41% and 48%, respectively. This effect was strongly amplified by 8MQPM, which reduced the tube length by 78% and brought back the branch points number to 77% (panel A, box d). The quantifications of tube total length and branch point number are reported in panels B and C, respectively.

### 3.7 Signal pathway investigation

**Figure 6:**
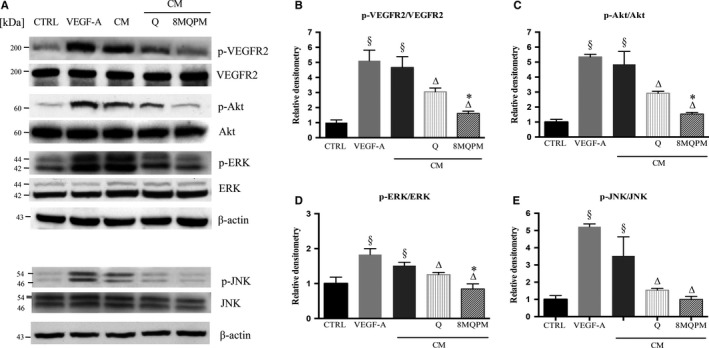
Effects of quercetin (Q) and 8MQPM on signal transduction. A representative blot is shown for p‐VEGFR2, p‐Akt, p‐ERK, p‐JNK and total expression (A). Densitometry analysis of phosphorylated/total protein ratio of each band (adu) was performed using Imagej software: p‐VEGFR2/VEGFR2 (B), p‐Akt/Akt (C), p‐ERK/ERK (D), p‐JNK/JNK (E). Values are expressed as the mean ± SD of three independent experiments (*n* = 3). ^∆^ *P* < 0.01 versus conditioned medium (CM)‐stimulated human retinal endothelial cells; ^*^ *P* < 0.01 versus Q. One‐way ANOVA followed by Tukey's test. VEGF, vascular endothelial growth factor; VEGFR‐2, VEGF receptor‐2

Experiments were performed in order to investigate the possible involvement of Q and 8MQPM in regulating the activation of VEGFR‐2 (Figure 6), the VEGF‐A receptor mainly involved in the intracellular signal transduction of the growth factor in endothelium.^41^ The VEGF‐A and the CM triggered the VEGFR‐2 phosphorylation by almost 5.1‐ and 4.6‐fold, respectively, at 15 minutes of incubation (ratios: p‐VEGFR‐2/VEGFR‐2 densitometry lectures; arbitrarily set equal to 1.0 in control cells). Both Q compounds were co‐incubated with CM: Q and 8MQPM prevented the receptor phosphorylation by 35% and 65%, respectively. All the treatments (VEGF‐A and CM stimulation, Q and 8MQPM in the presence of CM) had no effect on total VEGFR‐2. Quercetin and 8MQPM alone had no effect on both p‐VEGFR‐2 and VEGFR‐2 (data not shown).

In order to investigate the putative role of Q and 8MQPM in affecting the activity of well‐known proteins involved in the intracellular signalling of VEGF,^42^ the levels of expression/activation of Akt, ERK and JNK, were examined. In HREC, CM‐stimulated activations peaked at 15 minutes incubation (4.9‐, 1.5‐, 3.5‐fold increase for Akt, ERK 1/2 and JNK, respectively) in comparison with control cells. In the presence of CM, Q caused a significant reduction in almost 41%, 20% and 58% for p‐Akt, p‐ERK and p‐JNK, respectively. Interestingly, 8MQPM was more effective in reducing the phosphorylation levels (by 70%, 47% and 72%, for p‐Akt, p‐ERK and p‐JNK, respectively). These data shed light on the issue that Q and even more 8MQPM inhibit the primary HREC proliferation, migration, spatial organization and maturation by affecting Ras downstream cascade of MEK/ERK, MEK/JNK and PI3‐K/AKT pathways.

## 4 DISCUSSION

The imbalance among the molecules that coordinate the neoangiogenesis is often the cause of inflammatory, immune, infectious and malignant disorders.^38^ The newly formed blood vessels are fenestrated, permeable, tortuous and heterogeneous both in their structure and efficiency of perfusion.^43^ *Aberrant angiogenesis*, in fact, is responsible for several ocular diseases, such as choroidal subretinal neovascularization, retinopathy of prematurity, proliferative diabetic retinopathy^4^ and retinoblastoma.^44^, ^45^ The VEGF family (VEGFA, VEGFB, VEGFC, VEGFD and placental growth factor [PlGF]) and their cognate receptors are the leading molecular players in angiogenesis. The main mediators of tumour angiogenesis are both the two VEGFA isoforms, that is, the soluble VEGF121 and VEGF165, although the principal signalling tyrosine kinase receptor is the VEGF receptor 2 (VEGFR‐2; FLK, KDR in humans). For this reason, the inhibition of the binding of VEGFA with VEGFR‐2 is the goal‐target for biological cancer therapies. Moreover, there is demonstrated evidence that the inhibition of VEGFR2 not only blocks angiogenesis in tumours but it can also destroy the tumour vessels.^46^

In phase I‐ or II‐ clinical trials for cancer treatment, soluble decoy receptors, ligands sequesters,^47^ kinase activity inhibitors,^48^ several angiogenesis inhibitors were administered after their approval by the United States Food and Drug Administration.^49^ Unfortunately, serious side effects were observed with the currently available anti‐angiogenic drugs, which limited their continuous use.^43^ For these reasons, a growing interest in identifying natural health‐safe products is emerging. These novel phytochemicals contain a variety of anti‐angiogenic compounds, which could be used for developing anti‐angiogenic drugs.^50^

Flavonoids are a family of polyphenolic compounds extracted from fruits and plants. The basic flavonoid structure consists of two aromatic rings connected to a pyran ring (see Scheme 1 for the basic aglycon structure). Most flavonoids occur in plants as glycosides though some can also be present as free aglycones, that is, without attached sugars.^51^ Several evidences also show that Q and its analogues inhibit enzymes such as xanthine oxidase, lipoxygenase, cyclooxygenase and NADPH oxidase attenuating therefore the oxidative stress.^52^ Nowadays, Q and derivatives are considered attractive compounds for cancer prevention due to their anti‐proliferative and antioxidant abilities, and their role in the regulation of cell cycle, cell signalling and apoptosis, as demonstrated by several in vivo and in vitro studies.^53^ Quercetin also inhibits angiogenesis in human umbilical vein endothelial cells and zebrafish by suppressing the VEGF‐induced VEGFR2 phosphorylation, by the inhibition of kinases such as c‐Src, FAK, ERK, AKT, mTOR and S6K and by the induction of apoptosis.^54^

In the cancer treatment, many effective drugs are of natural origin,^50^ being modern medicines often very expensive, toxic and less effective in treating the disease. Several studies present in the literature are focused on the design of new Q derivatives with better performances in cancer treatment. For instance, it has recently been reported that Q and several synthetic derivatives show selective cytotoxicities in glioma cells, proving that the selective esterification and/or bromination of Q increase the toxicity of these polyphenols against cancer cells.^55^

In this study, we show the effects of Q and its synthetic derivatives 8MQPM, 6,8diBrQ and QD (see Scheme 1) on human primary endothelial cells isolated from retinal microvessels (HREC) as a BRB model system. This in vitro model could represent a starting point for future confirmations by using in vivo model systems. The cells were treated with CM collected from Y‐79 human retinoblastoma cell line, or VEGFA‐stimulated in order to reproduce the *angiogenic‐switch.*

The derivatives of Q, shown in Scheme 1, were designed in order to increase the lipophilicity of Q (8MQPM) or its toxicity (6,8‐diBrQ). On the other hand, QD (see Scheme 1) proved to be in a previous study,^32^ an effective antioxidant for prevention of age‐related macular degeneration and for this reason it was included in this study.

In dose‐ and time‐dependent evaluations, Q and all the derivatives represented in Scheme 1 caused a significant decrease in HREC viability at the highest doses used at 24 and 48 hours, showing, therefore, to exerting some toxicity on endothelial cells. On the other hand, at the lowest concentration of 25 μmol/L, 8MQPM was the less toxic and better tolerated compound whereas Q, 6,8‐diBrQ and QD showed a small but significant toxicity.

Quercetin and 8MQPM caused a significant decrease in retinoblastoma Y‐79 CM‐stimulated HREC proliferation, bringing back the cells to almost the control proliferation levels. On the contrary, 6, 8‐diBrQ and QD failed to attenuate the induced angiogenic effects. These data confirm the results of previous studies done in different areas and model systems.^56^, ^57^

The first step in angiogenesis takes place with the formation of new sprouts off from the existing vasculature, mediated by cell proliferation and tip cell migration.^58^ In wound‐healing assays, 8MQPM much more than Q (see Figure 2) decreased the HREC migration induced by retinoblastoma CM, whereas 6,8‐diBrQ and QD did not show any significant effects. Furthermore, 8MQPM and to a less extent Q were also able to inhibit the retinoblastoma‐stimulated tube formation (see Figure 4). The tube total length and the number of branch points from single HREC were both significantly decreased compared to cells treated with retinoblastoma CM or VEGFA. Interestingly, 8MQPM was able to bring back the branch points number to the control values (HREC not stimulated with CM or growth factor). In ex vivo rabbit aortic ring experiments, the incubation of the pieces with Q or 8MQPM in the presence of CM decreased significantly both the stimulated tube length and the branch points. Again, the effects observed with 8MQPM were overwhelming those observed with Q (see Figure 5).

The quantification of TEER through the endothelium monolayer isolated from anatomical barriers is a widely accepted technique to measure the integrity of tight junction systems. The measurement of TEER in our system showed that the barrier physical function, as expected, was heavily compromised because of the VEGF‐A and tumour CM treatments. The addition of Q caused, however, significant recoveries of TEER values at the first, second and third day of incubation. Surprisingly, 8MQPM was able to, almost completely, re‐establish the TEER to the control values, and hence conferred a strong protection against the collapsing effects of pro‐angiogenic environments on HREC. The other derivatives of Q (6,8‐diBrQ and QD) had no effects on TEER decrement.

**Figure 7:**
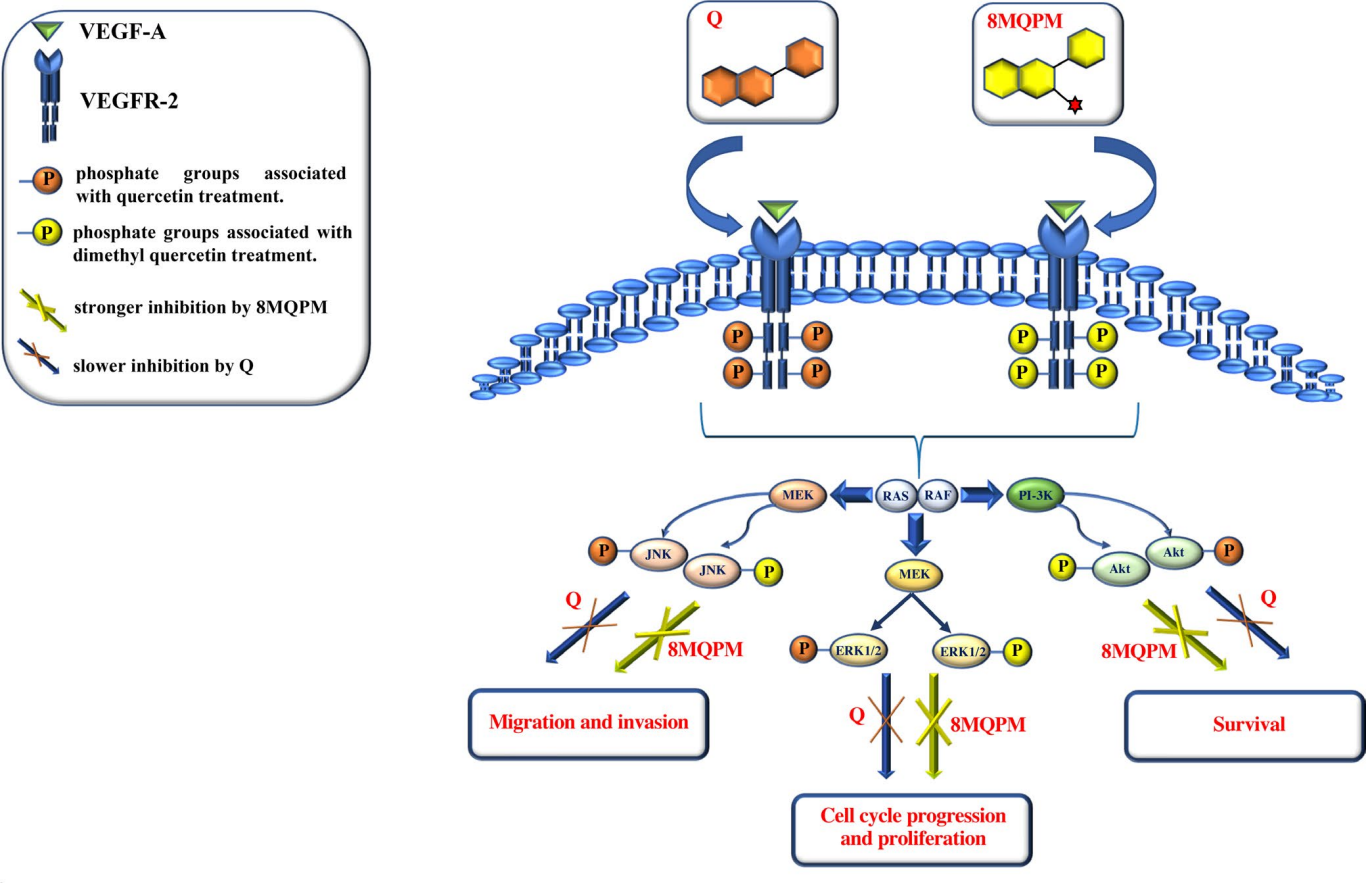
Inhibition of the angiogenic events, induced by VEGF‐A, with quercetin (Q) or 8MQPM. In the proposed model, Q and 8MQPM inhibit the VEGFR‐2 activation and its downstream cascade of events, eventually leading to the inhibition of ERK1/2, Akt and JNK activation/phosphorylation. There is therefore a counteraction on migration, invasion and morphogenesis of HREC. 8MQPM inhibits angiogenesis more effectively than the native Q. ERK, extracellular signal‐regulated kinase; JNK, c‐Jun N‐terminal kinase; VEGF, vascular endothelial growth factor; VEGFR‐2, VEGF receptor‐2

It was demonstrated that Q inhibits the VEGF‐induced migration, invasion, proliferation and tube formation in HUVECs through the inhibition of the AKT/mTOR/P70S6K pathway activation by VEGFR‐2 phosphorylation/activation. Moreover, it blocked the microvessel sprouting from rat aortic ring and CAM and inhibited cancer expansion and the related angiogenesis in human prostate xenograft mouse model.^25^ In our experiments, VEGFA and retinoblastoma CM strongly increased the VEGFR‐2 phosphorylation at Tyr1175 site. To understand the downstream molecular mechanism of the anti‐angiogenic ability of Q and 8MQPM, we also examined the signalling molecules and pathway involved in VEGFR‐2 endothelial cell response. VEGFR‐2‐stimulated activation of Akt, ERK and JNK transduce proliferation, migration, survival and the resistance to apoptosis in endothelial cells.^41^ In clinical trials, the successful of the anti‐angiogenic therapy often requires the simultaneous blockade of signalling downstream molecules from multiple pro‐angiogenic factor receptors.^59^ In our study, we found that 8MQPM blocked, much better than Q, the multiple downstream signalling components of VEGFR‐2, such JNK, ERK and Akt, suggesting that these molecules exerted their anti‐angiogenic activity by regulating the activation of VEGFR‐2‐mediated downstream signalling cascade in retinal endothelial cells subjected to a tumorigenic stimulus. These data confirm previous results obtained from other cell types and model systems.^60^ Moreover, it has been demonstrated, by docking simulations, that Quercetin compounds directly bind to the VEGFR2's active site, by forming four hydrogen bonds, with binding energies of −9.1 Kcal/mol.^61^ A proposed model of Q and 8MQPM inhibitory effect on retinoblastoma CM‐stimulated on angiogenesis is reported in Figure 7 (graphical abstract).

## 5 CONCLUSIONS

This study shows that some derivatives of Q exhibit divergent effects on tumour‐stimulated primary human retinal endothelium and on angiogenesis. We found that a permethylated form of Q, namely 8MQPM, more than the simple Q, is a potent inhibitor of angiogenesis both in vitro and ex vivo. These effects were not observed with other Q derivatives synthesized by us (6,8‐diBrQ and QD) which demonstrated to be toxic at the concentration used in the experiments and unable to inhibit the activation of VEGFR‐2/angiogenesis. The novelty of these results is that the treatment with 8MQPM and to a less extent with Q was able to inhibit the activation of VEGFR‐2, thereby suppressing the HREC proliferation, migration, spatial organization and Ras downstream cascade of MEK/ERK, MEK/JNK and PI3‐K/AKT pathways. Further investigations, by using ex vivo and in vivo models, are needed to confirm that 8MQPM might be a promising retinal medication.

## CONFLICTS OF INTEREST

The authors declare no conflict of interest.
